# Effect of acupuncture on somatic symptom disorder: a systematic review and meta-analysis

**DOI:** 10.3389/fmed.2025.1625230

**Published:** 2025-10-01

**Authors:** Feixue Zhao, Weiming Wang, Xinyao Feng, Jinyu Li, Weijuan Gang, Xianghong Jing, Huan Chen, Ying Liang

**Affiliations:** ^1^Peking University Sixth Hospital, Peking University, Beijing, China; ^2^Department of Acupuncture and Moxibustion, Guang’anmen Hospital, China Academy of Chinese Medical Sciences, Beijing, China; ^3^Institute of Acupuncture and Moxibustion, China Academy of Chinese Medical Science, Beijing, China; ^4^Key Laboratory of Mental Health, National Clinical Research Center for Mental Disorders, Ministry of Health, Institute of Mental Health, Beijing, China

**Keywords:** somatic symptom disorder, SSD, persistent somatoform pain disorder, PSPD, acupuncture, meta-analysis

## Abstract

**Objective:**

The rising prevalence of somatic symptom disorder (SSD) lacks specific treatment options. While acupuncture shows promise for mental health, its efficacy for SSD remains unclear. This systematic review and meta-analysis aims to clarify the evidence on acupuncture’s effectiveness for SSD.

**Methods:**

Eight databases including PubMed, Embase, Cochrane Library, Web of Science (WoS), China National Knowledge Internet (CNKI), etc. were searched from the inception to 15 March 2024. Randomized controlled trials (RCTs) that assessed the effect of acupuncture used alone or in combination with other therapies for SSD were included. Two independent reviewers performed study screening and data extraction. Risk of bias of included studies was assessed using Cochrane Risk-of-Bias (RoB) tool version 2. Meta-analysis was conducted where applicable.

**Results:**

Out of 9,526 articles, 5 studies with 376 patients were selected. Four of the studies showed the pooled estimates of mean difference in the change of HAMA scores between acupuncture plus paroxetine or duloxetine group and medication alone group were statistically significant at week 4 (−1.94, 95%CI: −3.71 to −0.17; *p* = 0.03) with borderline significance at week 6/8 (−3.17, 95%CI: −6.38 to 0.04; *p* = 0.05) from baseline. The pooled mean difference in change of numeric rating scale (NRS) score was not statistically significant between acupuncture plus duloxetine group and duloxetine alone group at week 2 (−1.25, 95%CI: −3.03 to 0.53; *p* = 0.17), 4 (−0.96; 95%CI: −2.30 to 0.38; *p* = 0.16), and 6/8 (−1.27, 95%CI: −3.81 to 1.26; *p* = 0.33) from baseline. Adverse event rates were comparable between acupuncture plus SSRI/SNRI and SSRI/SNRI alone, except in the comparison of floating acupuncture with placebo versus simulated floating acupuncture with duloxetine. All studies exhibited bias concerns or high risk of bias. Certainty of all outcomes was judged to be low or very low by using the Grading of Recommendations Assessment, Development and Evaluation (GRADE) approach.

**Conclusion:**

Our findings indicated potential added benefits of acupuncture combined with SSRI/SNRI for SSD-related anxiety, although effects on pain were inconsistent. High-quality RCTs with larger sample sizes are required to confirm acupuncture’s efficacy and safety for SSD.

**Systematic review registration:**

https://www.crd.york.ac.uk/PROSPERO/view/CRD42024537063, Identifier CRD42024537063.

## 1 Introduction

Somatic symptom disorder (SSD), also known as bodily distress disorder (BDD), is a mental illness in which patients persistently have one or more somatic symptoms that are distressing or result in significant impairment of daily life, accompanied by excessive or disproportionate attention related to the somatic symptoms and prominent anxiety and depression (1, 2).

The prevalence of SSD among general adult population varies substantially across region and settings (3). Cross-sectional studies reported that the prevalence of SSD was 4.5% in general population of Germany (4) and 33.8% in outpatient departments of general hospitals in China (5). Owing to the latest expansion of diagnostic criteria, the prevalence of SSD is anticipated to increase (1). According to a survey, the annual consumption of medical resources attributable to “somatization” in United States accounts for 16% of all healthcare expenditures, which is more than twice of other mental health disorders (6).

Current treatment challenges for SSD highlight the need for alternative interventions. Existing guidelines lack international consensus on SSD management (7), and no medications are specifically approved for SSD treatment (8). Psychotherapy, particularly cognitive behavioral therapy (CBT), is recommended as a first-line approach (9), yet its implementation is hindered by limited access to trained therapists, high costs, and low patient acceptance of psychological interventions (10–12). Pharmacological treatments, such as antidepressants, might exacerbate somatic symptoms or lead to adverse effects (9, 13), thereby reducing compliance and compromising long-term outcomes (14, 15). Therefore, it is imperative to develop alternative interventions for SSD with better efficacy and less side effect.

Acupuncture emerges as a promising adjunctive therapy due to its unique advantages in mental healthcare. As a non-pharmacological therapy, acupuncture exerts synergistic therapeutic effects through targeted regulation of key neurobiological pathways implicated in psychiatric pathophysiology, such as brain function network, neurotransmitter levels, neuroendocrine axis, neuroplasticity, anti-inflammatory, and other biological pathways (16). Growing evidence suggests that acupuncture can alleviate anxiety and depression with efficacy comparable to CBT (17), while exhibiting fewer side effects than conventional medications (18, 19), avoiding the accumulation of toxicity from drug metabolism as a physical stimulus.

Despite these mechanistic and tolerability advantages, the clinical application of acupuncture in SSD remains controversial. The 2020 meta-analysis (20) by Zhang et al. on acupuncture for somatoform disorders was limited by outdated diagnostic criteria and a narrow scope of outcomes. Recent randomized controlled trials (RCTs) have explored novel acupuncture protocols (e.g., floating acupuncture) and combination therapies, yet their findings have not been systematically evaluated. Therefore, this study aimed to update the literature and result of previous study to provide solid evidence for clinical practice and future research on SSD.

## 2 Methods

The Preferred Reporting Items for Systematic Reviews and Meta-analyses (PRISMA) was followed in reporting of this systematic review and meta-analysis (21). The protocol of this systematic review was registered in the PROSPERO database (registration number: CRD42024537063).

### 2.1 Search strategy

Literature search was conducted in eight databases, namely, PubMed, Embase, Cochrane Library, Web of Science (WoS), China National Knowledge Internet (CNKI), Wanfang Data, VIP Database for Chinese Technical Periodicals, and China Biology Medicine disc (CBM). The search period was from their inception to 15 March 2024. The main search terms include somatic symptom disorder, acupuncture, and randomized controlled trial. The search strategy was compiled by combining free words and subject headings and tailored according to the characteristics of each database. The references of previous reviews were also searched for relevant literature. The detailed search strategies are described in Supplementary material S1.

### 2.2 Inclusion and exclusion criteria

Studies that met all of the following criteria were included in this review: (a) patients were diagnosed as “somatic symptom disorder” or “bodily distress disorder (BDD)” according to the Diagnostic and Statistical Manual of Mental Disorders, 5th Edition (DSM-5 TM) or International Classification of Diseases 11th Revision (ICD-11), regardless of age, sex, and severity of the disease; (b) at least one group of interventions in the study was acupuncture alone or acupuncture in combination with other conservative or pharmacological treatments; (c) the type of study design was randomized controlled trial; (d) outcomes included, but not limited to, Hamilton Depression Scale (HAMD), the Hamilton Anxiety Scale (HAMA), Symptom Checklist-90 (SCL-90), etc.; (e) the language is limited to Chinese or English.

Studies with one of the following characteristics will be excluded: (a) patients had other types of mental or psychiatric disorders, or were lactating and pregnant women; (b) herbal medicine or moxibustion was included in any intervention arms, or comparison of interventions was made between different types of acupuncture, or different frequencies or protocol of the same type of acupuncture; (c) systematic reviews, secondary analyses of RCTs, and conference proceedings; (d) no required outcome data available for data analysis.

### 2.3 Study selection

After removal of duplications, titles and abstracts of the identified studies were independently reviewed by two reviewers (FZ and WW) against the inclusion and exclusion criteria of the review. Studies that were not relevant to the review were excluded. Full texts of the remaining studies were subsequently retrieved and reviewed by the two reviewers to identify eligible studies. Any disagreements were solved by senior reviewer (YL or HC).

### 2.4 Data extraction

The data were extracted from included studies according to a standardized form with the following information: author, year, country, diagnostic criteria, demographic characteristics of target population, intervention regimens of the experiment and control groups, time of follow-up, outcome measures, methods of statistics, and results. In addition, the number, type, and severity of adverse events were recorded. Two independent reviewers conducted the data extraction, and disagreements were completely discussed or solved by a senior reviewer.

### 2.5 Risk of bias assessment

The risk of bias assessment was conducted using Cochrane Risk-of-Bias (RoB) tool version 2. The risk of bias of included trials was assessed in each of the five domains, and an overall assessment of “high risk”, “some concerns”, or “low risk” was rated. The major domains of bias were as follows: randomization process, deviations from intended interventions, missing outcome data, measurement of the outcome, and selection of the reported result. Two reviewers independently assessed the risk of bias of included studies using RoB2. Any disagreement was resolved by discussion, with involvement of a third reviewer where necessary.

### 2.6 Certainty assessment of evidence

The Grading of Recommendations Assessment, Development and Evaluation (GRADE) approach was conducted to evaluate the certainty of evidence.

### 2.7 Statistical analysis

Continuous variables were presented as mean difference (MD) with standard deviation (SD), and dichotomous variables by risk ratio (RR) with 95% CI, respectively. Meta-analysis was conducted to present a pooled estimate of the treatment effect where similar study designs, intervention, and outcome measures were identified among studies. A fixed effect model was adopted for studies when heterogeneity was acceptable (I^2^ ≤ 50%), and a random effect model adopted when substantial heterogeneity was detected (I^2^>50%) in meta-analysis. Narrative analysis was performed for the data that could not be synthesized statistically. The RevMan V.5.4 was used for data analysis and synthesis.

## 3 Results

### 3.1 Characteristics of included studies

**Figure 1:**
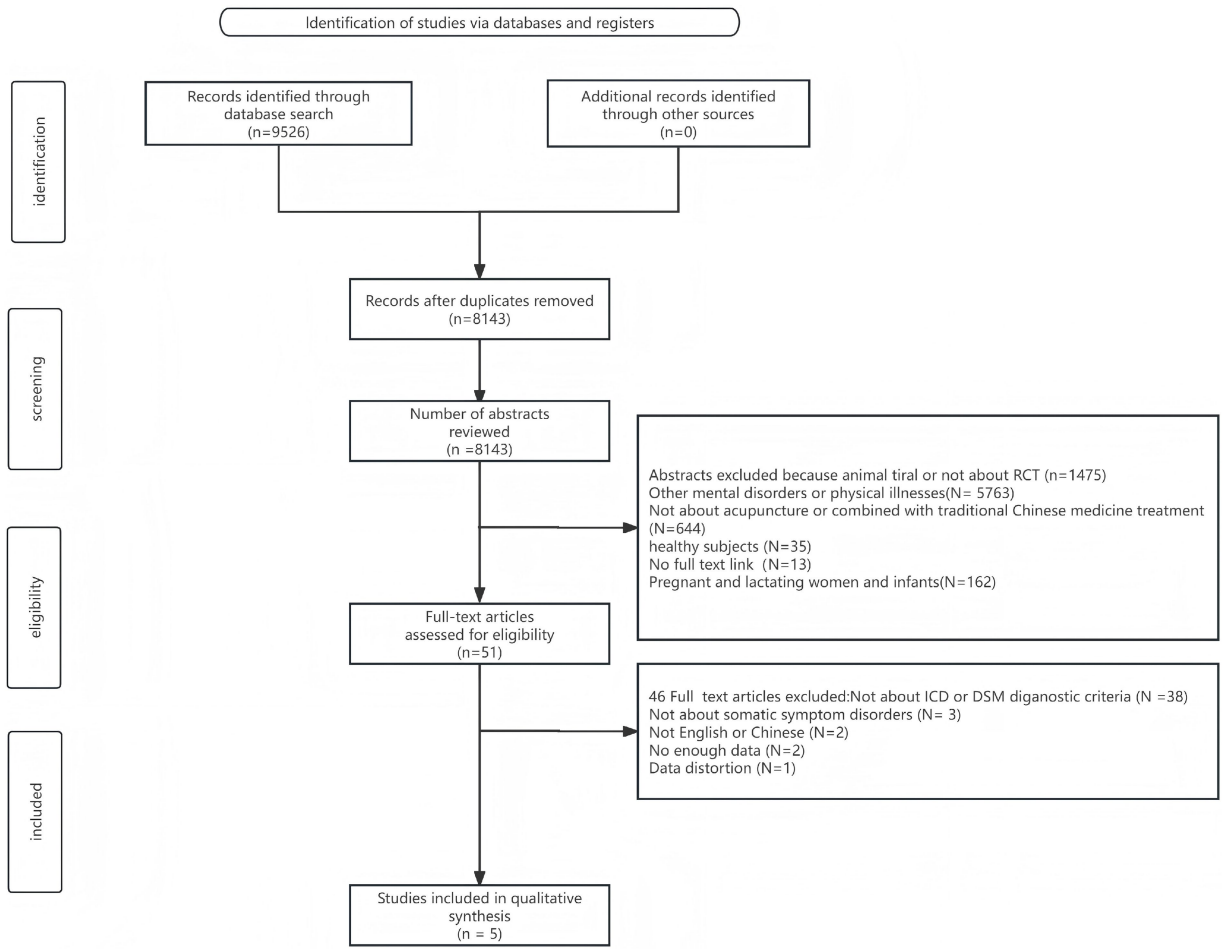
Study selection process.

The eight electronic databases were searched, a total of 9,526 articles were retrieved, and 1,383 duplications were removed. A total of 8,092 articles were excluded after screening by title and abstract according to eligibility criteria. Finally, 51 articles were screened by full-text reading, of which 46 studies were excluded, and 5 studies were eventually included. Figure 1 illustrates the study selection process.

The five studies included were all from China with a total of 376 patient samples (22–26). The studies by Chen (22), Sun et al. (23), Ma et al. (25), and Ren et al. (26) focused on patients with persistent somatoform pain disorder (PSPD, a subtype of SSD), while the other study (24) targeted general SSD patients. All studies reported that the baseline data were comparable. Four of the studies (22–25) used different types of acupuncture combined with antidepressants as the experimental group and antidepressants alone as the control group to evaluate the incremental effect of acupuncture for SSD patients. One study (26) used floating acupuncture combined with a placebo as the experimental group and simulated floating acupuncture combined with antidepressants as the control group. The treatment duration ranged from 6 to 8 weeks, and the reported outcomes included the Hamilton Anxiety Scale (HAMA), Hamilton Depression Scale (HAMD), numeric rating scale (NRS), the Symptom Checklist-90 (SCL-90), Pittsburgh Sleep Quality Index (PSQI), World Health Organization Quality of Life (WHOQOL), and Short-Form McGill Pain Scale (SF-MPQ), including Pain Rating Index (PRI), Visual Analog Scale (VAS), and Present Pain Intensity (PPI) (shown in Table 1).

**Table 1:** Characteristics of the five included studies.

Author, year, country	Sample size	Diagnostic criteria	Experiment interventions	Control intervention	Treatment duration and sessions	Follow-up duration	Outcomes
Chen (22), China	82 patients	PSPD in ICD-10	Acu + paroxetine 20 ~ 40 mg/d PC7, HT7, PC9, Ashi points Duration: N/M Frequency: N/M	Paroxetine 20 ~ 40 mg/d	8 weeks	N/M	HAMD-17 HAMA SF-MPQ (PPI) Adverse events: TESS
Sun et al. (23), China	60 patients	PSPD in ICD-10	EA + Duloxetine 40 mg/d PC7, HT7, PC6, Ashi points Duration: 30 min 6 times a week 36 times	Duloxetine 40 mg/d	6 weeks (6 sessions)	N/M	HAMD-2 4 HAMA-1 4 NRS
Liang and Liang (24), China	70 patients	Somatoform disorder in ICD-10	Acu + Duloxetine 60 ~ 120 mg/d BL13, BL14, BL15, BL18, BL20 Duration: 30 min once daily 28 times	Duloxetine 60 ~ 120 mg/d	4 weeks (1 sessions)	N/M	SCL-90
Ma et al. (25), China	94 patients	PSPD in ICD-10	FA + Duloxetine 40 ~ 60 mg/d Acupoint: the location of the pain Duration: needle manipulation ends in which pain disappears, and then, the needle is pulled out after 12 h Once every other day 28 times	Duloxetine 40 ~ 60 mg/d	8 weeks	N/M	NRS PSQI WHOQOL Adverse events: self-report
Ren et al. (26), China	70 patients	PSPD in ICD-10	FA + Placebo 40 ~ 60 mg/d Acupoint: the location of the pain Duration: needle manipulation ends in which pain disappears, and then, the needle is pulled out after 12 h Once every other day (5 times as a session, at 3 ~ 5d interval)	SFA + Duloxetine 40 ~ 60 mg/d	6 weeks	6 weeks	HAMD-17 HAMA SF-MPQ (PRI + VAS + PPI) Adverse events: TESS

Acu: acupuncture; EA: electroacupuncture; FA: floating acupuncture; HAMA: Hamilton Anxiety Scale; HAMD: Hamilton Depression Scale; NRS: numeric rating scale; PPI: Present Pain Intensity; PRI: Pain Rating Index; PSPD: Persistent somatoform pain disorder; PSQI: Pittsburgh Sleep Quality Index; SCL-90: the Symptom Checklist-90; SFA: Simulated Floating Acupuncture; SF-MPQ: Short-Form McGill Pain Scale; TESS: Treatment Emergent Symptom Scale; VAS: Visual Analog Scale; WHOQOL: World Health Organization Quality of Life; N/M: Not Mentioned; Acupuncture points named according to WHO guideline.

### 3.2 Risk of bias assessment

In terms of randomization process, Chen (22) and Sun et al. (23) mentioned the use of random number table method for randomization in the original article. The remaining three studies (24–26) only mentioned randomization without describing specific methods, so it was considered that these three studies may be randomized, but because there was no significant difference in baseline characteristics between the two groups of participants in the three studies, they were assessed as low risk.

Due to the nature of acupuncture, blinding is often difficult to implement in non-sham acupuncture-controlled trials. One study (26) used simulated floating acupuncture in the control group to blind the patients, but the acupuncturist could not be blinded, so the study was rated as some concern in terms of the deviation of intervention measures. The rest four studies (22–25) adopted medication only in the control group, and it was not possible to blind both patients and treatment providers; therefore, they were rated as high risk of bias under this domain.

For missing outcome data, no study reported missing data; therefore, low risk of bias was rated. For selection of the reported results, protocols of all five studies were not available publicly, so we were not sure whether there were unreported outcomes, and some concerns were rated.

**Figure 2:**
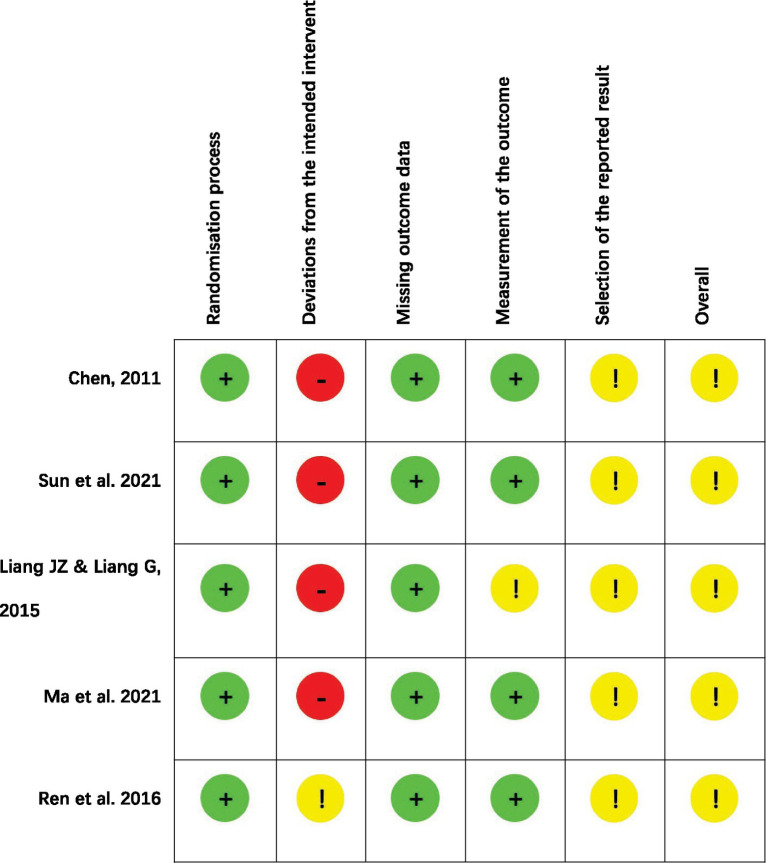
Risk of bias result for each included study.

**Figure 3:**
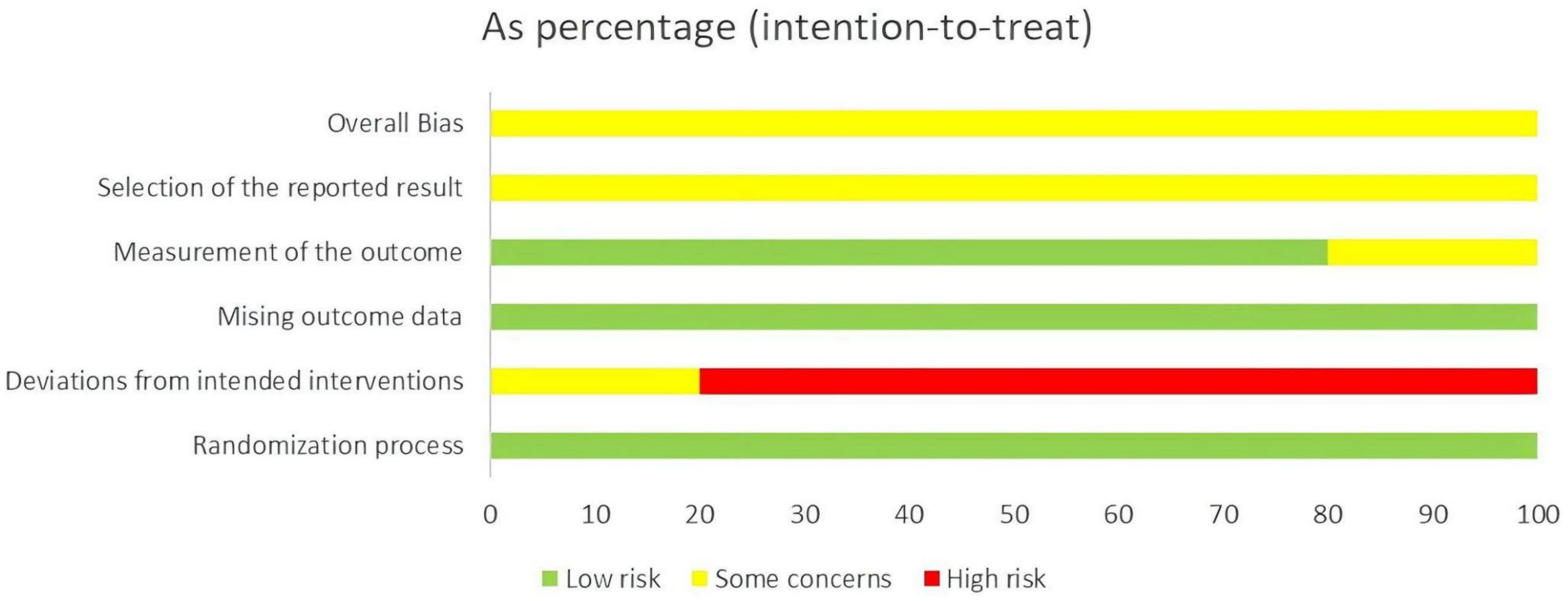
Summary of risk of bias result.

For the measurement of the outcome, the assessment criteria of Liang and Liang (24) were not clear, and the outcome measures used in the study were not specific for SSD and were assessed as some concern. Therefore, the overall risk of bias for all studies was rated as some concerns (shown in Figures 2, 3).

### 3.3 GRADE assessment

Three outcome measures were included in the GRADE assessment, and every outcome was reported by two trials. For HAMA scores, the evidence was rated as low certainty at 2 and 4 weeks (downgraded due to serious risk of bias and imprecision) and very low certainty at 6/8 weeks (further downgraded for inconsistency and indirectness). For NRS scores, evidence across all timepoints (2, 4, and 6/8 weeks) was assessed as very low certainty (downgraded for serious risk of bias, inconsistency, and imprecision). Adverse event data demonstrated moderate certainty (downgraded once for risk of bias). The full GRADE summary of findings is presented in Supplementary material S2.

### 3.4 Acupuncture plus antidepressant vs. antidepressant

Of the included studies, four compared the effect of acupuncture combined with antidepressant [selective serotonin reuptake inhibitor (SSRI) and serotonin and norepinephrine reuptake inhibitor (SNRI)] to antidepressant alone. Chen (22) (*n* = 82) compared acupuncture combined with paroxetine (SSRI) to paroxetine alone; Sun et al. (23) (*n* = 60) compared electroacupuncture combined with duloxetine (SNRI) to duloxetine alone; Liang and Liang (24) (*n* = 70) compared acupuncture combined with duloxetine (SNRI) to duloxetine alone; Ma et al. (25) (*n* = 94) compared floating acupuncture combined with duloxetine (SNRI) to duloxetine alone (shown in Table 1).

#### 3.4.1 HAMA

Chen (22) and Sun et al. (23) used HAMA to evaluate the improvement of anxiety symptoms in patients with SSD. Chen (22) reported that the mean difference in change of HAMA was not statistically significant between the experimental and control groups at weeks 2 (−0.30, 95%CI: −2.25 to 1.65; *p* = 0.76), 4 (−1.70, 95%CI: −3.98 to 0.58; *p* = 0.15), and 8 (−1.70, 95%CI: −3.51 to 0.11; *p* = 0.07) from baseline, respectively (shown in Table 2).

**Table 2:** Summary of outcomes.

Studies	Sample size	Outcome measurement		Experiment group			Control group			Between-group difference	
				Baseline	Post-treatment	Changes	Baseline	Post-treatment	Changes	MD/RR (95%CI)	*p*-value
Chen (22)	43/39	HAMD-17 [Mean ± SD]	2 W	26.2 ± 3.7	21.5 ± 3.6	−4.7 ± 3.69	23.1 ± 5.6	21.7 ± 5.5	−1.4 ± 5.60	−3.30 [−5.37, −1.23]	0.0021
			4 W	–	13.6 ± 4.8	−12.6 ± 4.40	–	17.2 ± 4.6	−5.9 ± 5.22	−6.70 [−8.90, −4.50]	0.0000
			8 W	–	8.7 ± 5.6	−17.5 ± 4.98	–	11.5 ± 0.5	−11.6 ± 5.34	−5.90 [−8.14, −3.66]	0.0000
		HAMA [Mean ± SD]	2 W	18.2 ± 3.5	15.3 ± 3.7	−2.9 ± 3.64	19.1 ± 5.2	16.5 ± 5.0	−2.6 ± 5.16	−0.30 [−2.25, 1.65]	0.7601
			4 W	–	10.6 ± 5.7	−7.6 ± 5.02	–	13.2 ± 5.6	−5.9 ± 5.47	−1.70 [−3.98, 0.58]	0.1461
			8 W	–	7.7 ± 3.6	−10.5 ± 3.59	–	10.3 ± 3.5	−8.8 ± 4.63	−1.70 [−3.51, 0.11]	0.0655
		SF-MPQ (PPI) [Mean ± SD]	2 W	3.2 ± 0.7	2.5 ± 0.6	−0.7 ± 0.65	3.1 ± 0.8	2.7 ± 0.5	−0.4 ± 0.68	−0.30 [−0.59, −0.01]	0.0445
			4 W	–	1.5 ± 0.8	−1.7 ± 0.75	–	2.2 ± 0.6	−0.9 ± 0.71	−0.80 [−1.12, −0.48]	0.0000
			8 W	–	0.8 ± 0.6	−2.4 ± 0.65	–	1.5 ± 0.5	−1.6 ± 0.68	−0.80 [−1.09, −0.51]	0.0000
		TESS (case)	Nausea	–	1	–	–	–	–	–	–
			Low appetite	–	–	–	–	2	–	–	–
			Drowsy	–	1	–	–	1	–	–	–
			Thirst	–	1	–	–	–	–	–	–
			Constipation	–	–	–	–	1	–	–	–
			Total	–	3	–	–	3	–	–	0.57
Sun et al. (23) (PSPD)	30/30	HAMD −2 4 [Mean ± SD]	2w	28.3 ± 6.6	26.1 ± 5.7	−2.2 ± 6.78	28.3 ± 6.6	25.0 ± 5.5	−3.3 ± 6.69	1.10 [−2.31, 4.51]	0.5295
			4w	–	19.2 ± 4.9	−9.1 ± 6.46	–	22.9 ± 4.8	−5.4 ± 6.42	−3.70 [−6.96, 0.44]	0.0300
			6w	–	16.2 ± 4.1	−12.1 ± 6.22	–	21.9 ± 5.0	−6.4 ± 6.49	−5.70 [−8.92, −2.48]	0.0010
		HAMA −1 4 [Mean ± SD]	2w	18.9 ± 4.8	18.7 ± 5.3	−0.2 ± 5.55	19.7 ± 5.5	19.2 ± 5.0	−0.5 ± 5.77	0.30 [−2.56, 3.16]	0.8381
			4w	–	12.7 ± 5.3	−6.2 ± 5.55	–	15.8 ± 4.5	−3.9 ± 5.54	−2.30 [−5.11, 0.51]	0.1136
			6w	–	9.1 ± 5.1	−9.8 ± 5.43	–	14.9 ± 4.7	−4.8 ± 5.63	−5.00 [−7.80, −2.20]	0.0009
		NRS [Mean ± SD]	2w	6.4 ± 2.1	4.1 ± 1.9	−2.3 ± 2.20	5.9 ± 1.7	5.8 ± 2.0	−0.1 ± 2.04	−2.20 [−3.27, −1.13]	0.0002
			4w	–	3.2 ± 1.5	−3.2 ± 2.03	–	4.4 ± 1.8	−1.5 ± 1.92	−1.70 [−2.70, −0.70]	0.0015
			6w	–	2.2 ± 1.7	−4.2 ± 2.11	–	4.3 ± 1.9	−1.6 ± 1.98	−2.60 [−3.64, −1.56]	0.0000
Liang and Liang (24)	35/35	SCL −90 [Mean ± SD]	4w	222.30 ± 47.35	123.44 ± 14.07	−98.86 ± 43.67	227.60 ± 43.57	159.79 ± 19.27	−67.81 ± 39.98	−31.05[−50.66, −11.44]	0.0028
Ma et al. (25) (PSPD)	47/47	NRS [Mean ± SD]	1w	7.18 ± 1.76	5.09 ± 1.69	−2.09 ± 1.89	7.59 ± 1.83	5.87 ± 1.78	−1.72 ± 1.98	−0.37 [−1.15,0.41]	0.3565
			2w	–	4.12 ± 1.52	−3.06 ± 1.81	–	4.91 ± 1.61	−2.68 ± 1.98	−0.38 [−1.15,0.39]	0.3340
			4w	–	2.16 ± 0.84	−5.02 ± 1.62	–	2.90 ± 0.95	−4.69 ± 1.69	−0.33 [−1.00,0.34]	0.3364
			8w	–	0.95 ± 0.43	−6.23 ± 1.64	–	1.37 ± 0.52	−6.22 ± 1.69	−0.01 [−0.68, 0.66]	0.9768
		PSQI [Mean ± SD]	1w	16.78 ± 2.97	12.12 ± 2.56	−4.66 ± 3.05	16.96 ± 2.92	14.05 ± 2.81	−2.91 ± 3.14	−1.75 [−3.00, −0.50]	0.0074
			2w	–	9.14 ± 2.11	−7.64 ± 2.87	–	11.09 ± 2.35	−5.87 ± 2.93	−1.77 [−2.94, −0.60]	0.0039
			4w	–	6.92 ± 2.03	−9.86 ± 2.85	–	9.45 ± 2.15	−7.51 ± 2.85	−2.35 [−3.50, −1.20]	0.0001
			8w	–	4.81 ± 1.67	−11.97 ± 2.76	–	6.34 ± 1.83	−10.62 ± 2.85	−1.35 [−2.48, −0.22]	0.0218
		WHOQOL [Mean ± SD]	Physiological health	58.29 ± 6.27	81.34 ± 7.98	23.05 ± 7.94	58.97 ± 6.12	76.98 ± 7.84	18.01 ± 7.78	5.04 [1.86, 8.22]	0.0025
			Psychological status	59.34 ± 6.38	82.34 ± 8.10	23 ± 8.06	60.32 ± 6.72	77.83 ± 7.92	17.51 ± 8.08	5.49 [2.23, 8.75]	0.0014
			Social relationships	61.23 ± 6.93	83.22 ± 8.43	21.99 ± 8.51	62.09 ± 6.89	79.14 ± 7.92	17.05 ± 8.68	4.94 [1.46, 8.42]	0.0065
			Environment	62.75 ± 6.90	80.62 ± 7.84	17.87 ± 8.11	63.58 ± 6.89	75.07 ± 7.42	11.49 ± 7.85	6.38 [3.15, 9.61]	0.0002
		Adverse reactions case	Nausea and vomiting	–	2	–	–	2	–	–	–
			Thirst	–	2	–	–	1	–	–	–
			Constipation	–	2	–	–	1	–	–	–
			Low appetite	–	2	–	–	1	–	–	–
			Total	–	8	–	–	5	–	–	–
Ren et al. (26) (PSPD)	36/34	HAMD −17 [Mean ± SD]	1w	16.31 ± 2.29	12.11 ± 2.00	−4.2 ± 2.36	15.97 ± 2.39	14.16 ± 2.29	−1.81 ± 2.37	−2.39 [−3.50, −1.28]	0.0001
			2w	–	11.14 ± 2.53	−5.17 ± 2.65	–	10.94 ± 2.51	−5.03 ± 2.49	−0.14 [−1.34, 1.06]	0.8207
			4w	–	10.17 ± 3.02	−6.14 ± 2.97	–	10.53 ± 2.86	−5.44 ± 2.70	−0.70 [−2.03, 0.63]	0.3067
			6w	–	8.88 ± 3.42	−7.43 ± 3.27	–	8.72 + 3.60	−7.25 ± 3.21	−0.18 [−1.70, 1.34]	0.8171
		HAMA [Mean ± SD]	1w	15.86 ± 2.37	12.06 ± 2.29	−3.8 ± 2.55	15.81 ± 2.21	14.31 ± 2.13	−1.5 ± 2.20	−2.30 [−3.41, −1.19]	0.0001
			2w	–	11.31 ± 2.71	−4.55 ± 2.80	–	11.28 ± 2.67	−4.53 ± 2.51	−0.02 [−1.26, 1.22]	0.9710
			4w	–	10.28 ± 3.27	−5.58 ± 3.18	–	11.00 ± 2.75	−4.81 ± 2.56	−0.77 [−2.12, 0.58]	0.2701
			6w	–	8.72 ± 3.87	−7.14 ± 3.64	–	8.72 ± 4.10	−7.09 ± 3.59	−0.05 [−1.74,1.64]	0.9541
		PRI [Mean ± SD]	Immediately	8.64 ± 2.42	7.39 ± 1.70	−1.25 ± 2.34	8.85 ± 2.38	8.71 ± 2.14	−0.14 ± 2.31	−1.11 [−2.20, −0.02]	0.0499
			1w	–	7.06 ± 1.47	−1.58 ± 2.27	–	8.18 ± 1.53	−0.67 ± 2.13	−0.91 [−1.94,0.12]	0.0887
			2w	–	6.33 ± 1.79	−2.31 ± 2.37	–	6.26 ± 1.48	−2.59 ± 2.12	0.28 [−0.77, 1.33]	0.6048
			4w	–	5.25 ± 1.34	−3.39 ± 2.25	–	5.44 ± 1.44	−3.41 ± 2.12	0.02 [−1.00, 1.04]	0.9696
			6w	–	4.94 ± 1.19	−3.7 ± 2.23	–	5.06 ± 1.25	−3.79 ± 2.10	0.09 [−0.92, 1.10]	0.8627
		VAS [Mean ± SD]	Immediately	5.56 ± 0.94	5.08 ± 0.81	−0.48 ± 0.96	5.71 ± 0.97	5.68 ± 0.94	−0.03 ± 0.96	−0.45[−0.90, −0.00]	0.0541
			1w	–	4.31 ± 0.95	−1.25 ± 1.04	–	5.18 ± 0.94	−0.53 ± 0.96	−0.72[−1.19, −0.25]	0.0037
			2w	–	3.89 ± 0.78	−1.67 ± 0.95	–	4.06 ± 0.69	−1.65 ± 0.87	−0.02 [−0.45,0.41]	0.9272
			4w	–	3.47 ± 0.88	−2.09 ± 1.00	–	3.47 ± 0.96	−2.24 ± 0.97	0.15 [−0.31, 0.61]	0.5266
			6w	–	3.17 ± 0.81	−2.39 ± 0.96	–	3.38 ± 0.74	−2.33 ± 0.88	−0.06 [−0.49,0.37]	0.7864
		PPI [Mean ± SD]	Immediately	2.86 ± 0.35	2.17 ± 0.61	−0.69 ± 0.57	2.88 ± 0.33	2.82 ± 0.46	−0.06 ± 0.42	−0.63[−0.86, −0.40]	0.0000
			1w	–	1.97 ± 0.65	−0.89 ± 0.60	–	2.38 ± 0.55	−0.5 ± 0.49	−0.39[−0.65, −0.13]	0.0041
			2w	–	3.89 ± 0.78	1.03 ± 0.72	–	1.91 ± 0.67	−0.97 ± 0.59	2.00 [1.69, 2.31]	0.0000
			4w	–	1.69 ± 0.75	−1.17 ± 0.69	–	1.76 ± 0.74	−1.12 ± 0.65	−0.05 [−0.36,0.26]	0.7563
			6w	–	1.64 ± 0.72	−1.22 ± 0.66	–	1.74 ± 0.83	−1.14 ± 0.73	−0.08 [−0.41,0.25]	0.6318
		TESS (case)	Nausea	–	1	–	–	7	–	–	–
			Constipation	–	2	–	–	4	–	–	–
			Thirst	–	0	–	–	4	–	–	–
			Drowsy	–	0	–	–	2	–	–	–
			Total	–	3	–	–	17	–	–	0.0001

In Sun et al. (23)’s study, the between-group difference in the change of HAMA scores after 6 weeks of treatment in the experimental group was significantly higher than that in the control group (−5.00, 95% CI: −7.80 to −2.20; *p* = 0.0009) from baseline; however, the mean difference was not significant at weeks 2 (0.30, 95% CI: −2.56 to 3.16; *p* = 0.84) and 4 (−2.3, 95% CI: −5.11 to 0.51; *p* = 0.11) (shown in Table 2).

**Figure 4:**
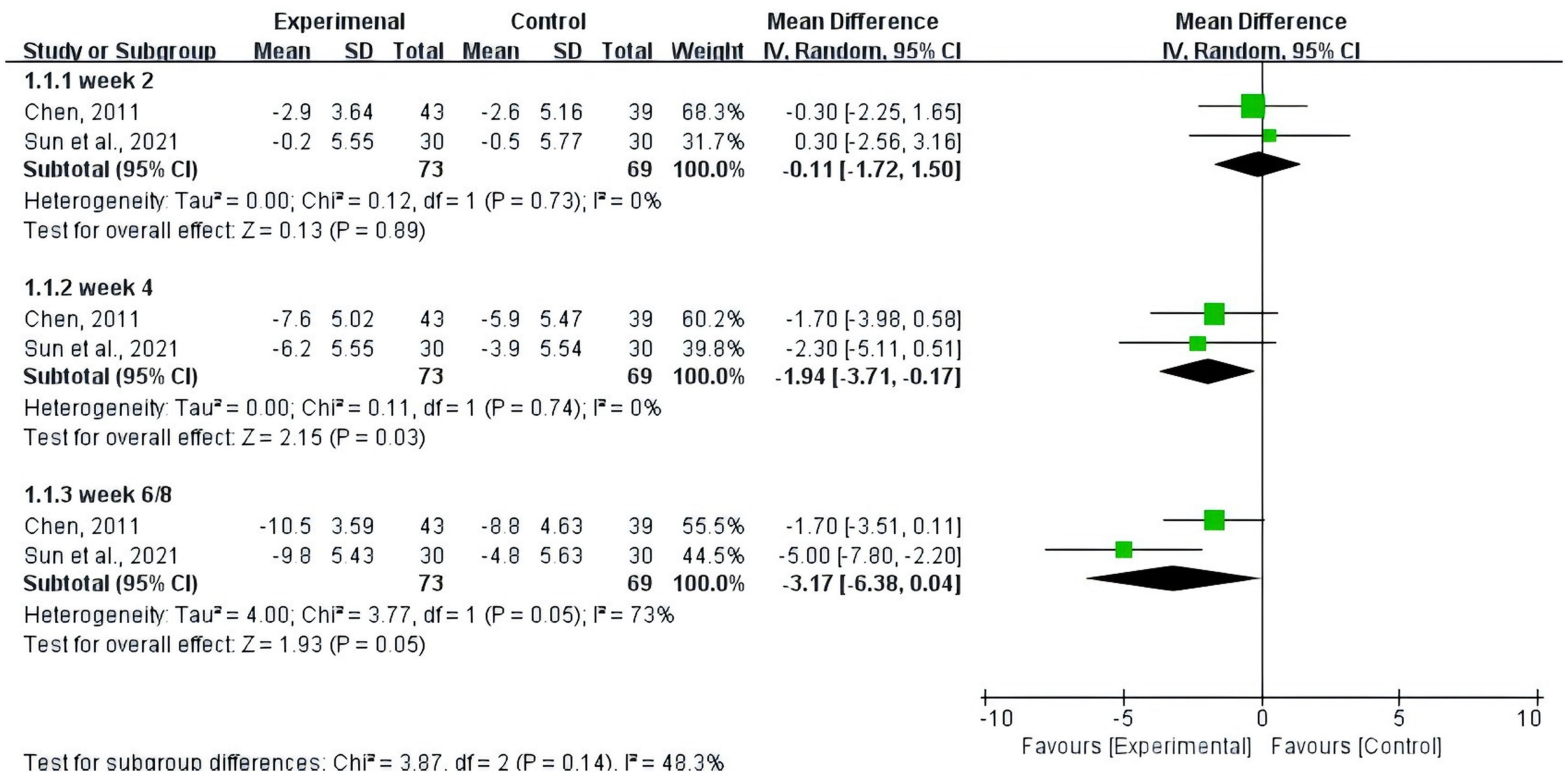
Pooled estimate on mean difference in the change of HAMA scores between the experiment and control groups by different follow-up time.

The pooled estimate showed that the change of HAMA scores in acupuncture plus paroxetine or duloxetine group was significantly greater than that in the control group at week 4 (−1.94, 95%CI: −3.71 to −0.17; *p* = 0.03) from baseline. However, the between-group difference at 6/8 weeks (−3.17, 95%CI: −6.38 to 0.04; *p* = 0.05) indicated a borderline significance. The mean difference between the two groups was not statistically significant at week 2 (−0.11, 95%CI: −1.72 to 1.50; *p* = 0.89) (shown in Figure 4).

#### 3.4.2 HAMD

Chen (22) and Sun et al. (23) reported HAMD scores, using HAMD-17 scale and the HAMD-24 scale, respectively. Due to the use of different measurement tools, data synthesis was not performed (shown in Table 2).

Chen (22) revealed a statistically significant difference in the change of HAMD scores at weeks 2 (−3.3, 95%CI: −5.37 to −1.23; *p* = 0.002), 4 (−6.70, 95%CI: −8.90 to −4.50; *p* < 0.0001), and 8 (−5.9, 95%CI: −8.14 to −3.66; *p* < 0.0001) from baseline between the experiment and control groups (shown in Table 2).

Sun et al. (23) reported that the difference in change of HAMD between the experiment and control groups was not significant at week 2 from baseline (1.10, 95%CI: −2.31 to 4.51; *p* = 0.53); however, they turned significant at weeks 4 (−3.7, 95%CI: −6.96 to −0.44; *p* = 0.03) and 6 (−5.70, 95%CI: −8.92 to −2.48; *p* = 0.001) from baseline, respectively (shown in Table 2).

#### 3.4.3 Pain assessment

The studies of Chen (22), Sun et al. (23), and Ma et al. (25) used NRS or Present Pain Intensity (PPI) as pain assessment outcomes, both of which are tools for measuring pain intensity in the form of questionnaire, with higher score indicating severer pain (27–29).

Chen (22) reported significant differences in the change of PPI scores between the experiment and control groups at weeks 2 (−0.3, 95%CI: −0.59 to −0.01; *p* = 0.04), 4 (−0.8, 95%CI: −1.12 to −0.48; *p* < 0.0001), and 8 (−0.8, 95%CI: −1.09 to −0.51; *p* < 0.0001) from baseline (shown in Table 2).

In Sun et al.’s (23) study, the NRS scores of the two groups showed a descending trend after treatment, and the between-group differences in the change of NRS scores were statistically significant at weeks 2 (−2.2, 95%CI: −3.27 to −1.13; *p* = 0.0002), 4 (−1.7, 95%CI: −2.70 to −0.70; *p* = 0.0015), and 6 (−2.6, 95%CI: −3.64 to −1.56; *p* < 0.0001) from baseline, respectively (shown in Table 2).

Ma et al. (25) showed that there was no significant difference in the change of NRS score between the two groups at weeks 1 (−0.37, 95%CI: −1.15 to 0.41; *p* = 0.36), 2 (−0.38, 95%CI: −1.15 to 0.39; *p* = 0.33), 4 (−0.33, 95%CI: −1.00 to 0.34; *p* = 0.34), and 8 (−0.01, 95%CI: −0.68 to 0.66; *p* = 0.98) from baseline, respectively (shown in Table 2).

**Figure 5:**
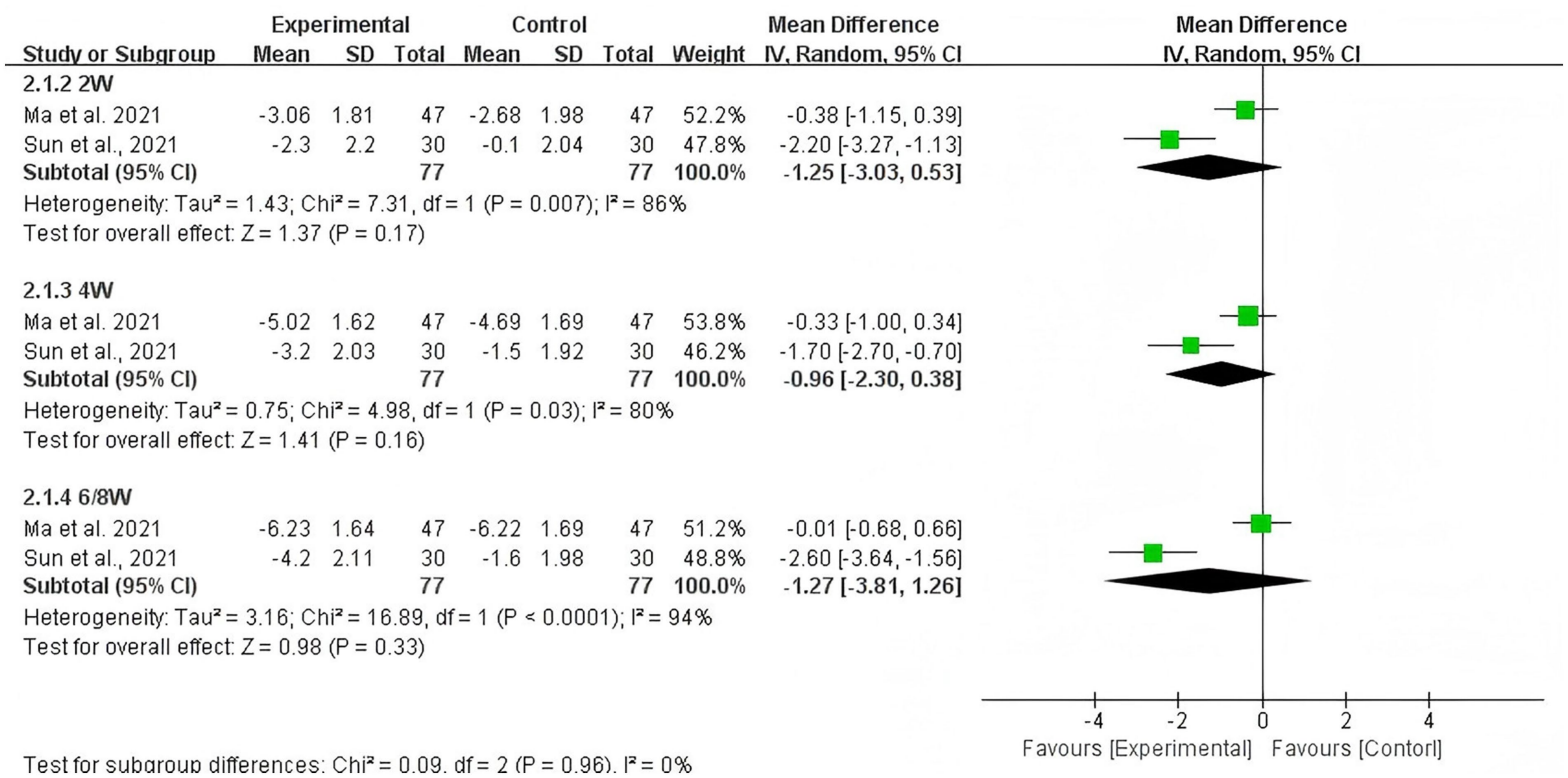
Pooled estimate on mean difference in the change of NRS scores between the experiment and control groups by different follow-up time.

The pooled estimate showed that the difference in change of NRS score was not statistically significant between acupuncture plus duloxetine group and duloxetine alone group at weeks 2 (−1.25, 95% CI: −3.03 to 0.53; *p* = 0.17), 4 (−0.96; 95%CI: −2.30 to 0.38; *p* = 0.16), and 6/8 (−1.27, 95%CI: −3.81 to 1.26; *p* = 0.33) from baseline, respectively (shown in Figure 5).

#### 3.4.4 SCL-90

Only Liang and Liang (24) evaluated the severity of somatic symptoms using SCL-90, and the results showed that the between-group difference in the change of SCL-90 scores at week 4 from baseline was statistically significant (−31.05, 95%CI: −50.66 to −11.44; *p* = 0.003) (shown in Table 2).

#### 3.4.5 Quality of sleep

In Ma et al.’s (25) study, the effect of floating acupuncture combined with duloxetine on sleep quality in PSPD patients was evaluated by PSQI, with higher score indicating worse sleep quality. It showed that there was statistically significant difference in the change of PSQI between the experiment and control groups at weeks 1 (−1.75, 95%CI: −3.00 to −0.50; *p* = 0.007), 2 (−1.77, 95%CI: −2.94 to −0.60; *p* = 0.004), 4 (−2.35, 95%CI: −3.50 to −1.20; *p* = 0.0001), and 8 (−1.35, 95%CI: −2.48 to −0.22; *p* = 0.02) from baseline, respectively (shown in Table 2).

#### 3.4.6 Quality of life

Ma et al. (25) used the WHOQOL to evaluate the quality of life of the two groups before and after treatment, including physical health, psychological state, social relationships, and environment. A higher score indicates a better quality of life (30). The mean difference in change of WHOQOL was statistically significant between the experiment and control groups in physical health (5.04, 95%CI: 1.86 to 8.22; *p* = 0.003), psychological state (5.49, 95%CI: 2.23 to 8.75; *p* = 0.001), social relationships (4.49, 95%CI: 1.46 to 8.42; *p* = 0.07), and environment (6.38, 95%CI: 3.15 to 9.61; *p* = 0.0002) at week 8 from baseline (shown in Table 2).

### 3.5 Floating acupuncture plus placebo vs. simulated floating acupuncture plus duloxetine

The study of Ren et al. (26) (*n* = 70) compared the effect of floating acupuncture combined with placebo to simulated floating acupuncture combined with duloxetine in patients with SSD for 6 weeks.

The results showed that the difference in change of HAMA from baseline between the experiment and control groups at week 1 (−2.30, 95%CI: −3.41 to −1.19; *p* = 0.0001) was statistically significant; however, the difference was not significant at weeks 2 (−0.02, 95%CI: −1.26 to 1.22; *p* = 0.97), 4 (−0.77, 95%CI: −2.12 to 0.58; *p* = 0.27), and 8 (−0.05, 95%CI: −1.74 to 1.64; *p* = 0.95), respectively. Similarly, the difference in change of HAMD from baseline was statistically significant between the experiment and control groups at week 1 (−2.39, 95%CI: −3.50 to −1.28; *p* = 0.0001) but not significant at weeks 2 (−0.14, 95%CI: −1.34 to 1.06; *p* = 0.82), 4 (−0.70, 95%CI: −2.03 to 0.63; *p* > 0.31), and 8 (−0.18, 95%CI: −1.07 to 1.34; *p* = 0.82) after treatment (shown in Table 2).

SF-MPQ (PRI, PPI, and VAS) was used to evaluate the effect of floating acupuncture for PSPD patients. The difference in change of PRI (−1.11, 95%CI: −2.20 to −0.02; *p* = 0.05) and PPI (−0.63, 95%CI: −0.86 to −0.40; *p* = 0.0000) between the two groups was statistically significant after treatment, indicating a better effect on pain relief in the experiment group. The difference in change of VAS score (−0.72, 95%CI: −1.19 to −0.25; *p* = 0.004) and PPI score (−0.39, 95%CI: −0.65 to −0.13; *p* = 0.004) was statistically significant between the two groups at week 1 after treatment but was not significant between the two groups at other time points (*p* > 0.05) (shown in Table 2).

### 3.6 Adverse effects

Of the five studies included, only three studies reported adverse effects. Chen’s (22) and Ren et al.’s (26) studies reported adverse events in the form of Treatment Emergent Symptom Scale (TESS) score, while Ma et al. (25) reported adverse effect through self-report (shown in Table 2).

Chen (22) reported that, in the early stage of study, there were two cases of nausea, one case of drowsiness, and one case of dry mouth in the experiment group, while there were two cases of nausea, two cases of anorexia, one case of drowsiness, and one case of constipation in the control group. After 8 weeks of treatment, the above adverse events were alleviated or disappeared.

Ma et al. (25) reported nausea and vomiting, dry mouth, constipation, and decreased appetite. However, there was no significant difference in the incidence of adverse events between the two groups (Fisher’s exact test, *p* = 0.57).

Ren et al. (26) reported 3 cases (8.3%) of adverse events in the experiment group and 17 cases (50.0%) in the control group, and difference in the incidence of adverse events between the two groups was statistically significant (Fisher’s exact test, *p* = 0.0001). Similarly, most of the adverse events, which were mild or moderate, occurred in early stages of treatment and alleviated or disappeared later. No serious adverse events were observed.

## 4 Discussion

The results showed that the pooled difference in change of HAMA scores in patients with SSD between acupuncture plus SSRI/SNRI group and SSRI/SNRI alone group was significant at week 4 from baseline, whereas the between-group difference of HAMA at 6–8 weeks only indicated a borderline significance. The pooled difference in pain assessment using NRS was not statistically significant between the two groups at weeks 2, 4, and 6/8 from baseline. Among three studies reporting adverse events, the incidences of adverse events were not significantly different between acupuncture plus SSRI/SNRI group and SSRI/SNRI alone group; however, it was not the case between floating acupuncture plus placebo group and simulated floating acupuncture plus duloxetine group. The overall risk of bias of included studies was rated as some concerns.

The result of this study is basically consistent with that of Zhang et al. (20), showing that the combination of acupuncture and medication had greater improvement than medication alone on HAMA score for patient with SSD; however, the combination of acupuncture and medication was not superior to medication alone in terms of VAS score for pain.

As there is no consensus on minimal clinically significant difference (MCID) of HAMA, one randomized controlled trial (*n* = 64) (31) on acupuncture for anxiety among patients with Parkinson disease used 4 as MCID based on anchor-based method. In reference to this value, the reduction of HAMA score in both acupuncture plus SSRI/SNRI group and SSRI/SNRI group reached MCID after 4 weeks of treatment in both Chen’s (22) and Sun et al.’s (23) studies and were even enlarged (but not significant) in both studies at week 6/8. This aligns with the pharmacokinetic profiles of paroxetine and duloxetine, both of which usually reach a target dose approximately 4 weeks of treatment and a full anxiolytic effect at 6/8 weeks (32–36). Although the mean between-group difference did not reach MCID at any time point in meta-analysis, it reached 5 points in Sun et al.’s (23) study at week 6 of treatment. This may be associated with electroacupuncture used in this study, which enhances the expression of hippocampal brain-derived neurotrophic factor (BDNF) and reshapes the functional connectivity of the prefrontal-limbic system through quantifiable electrical stimulation parameters and sustained duration, forming a multi-level anti-anxiety mechanism by promoting neuroregeneration and plasticity and restoring the balance of emotional regulation circuits, thereby demonstrating superior rapidity and sustainability in clinical anti-anxiety effects compared with traditional acupuncture (37–40). However, due to difference in target population in the RCT and this review, the benefit of acupuncture observed in review may be overestimated. In addition, it is notable that the overall risk of bias of included studies is of some concerns, which would undermine the reliability of the result; therefore, the interpretation of the effect of the combined therapy on HAMA should be cautious.

Pain is a major type of somatic symptoms for patient with SSD, and studies have proved that acupuncture has satisfactory analgesic effect for pain with less side effects (41–43). However, the analgesic effect observed across studies included was inconsistent and the pooled difference in pain assessment using NRS was not statistically significant between acupuncture plus SSRI/SNRI and SSRI/SNRI alone group through weeks 2–8. This may be associated with relatively mild severity of pain of patients at baseline in all included studies, as well as the varied selection of acupoints. Some types of SSRI/SNRI could also provide analgesic effect, such as duloxetine, which may narrow the difference in pain relief between the experiment and control groups (35). It is also interesting to see that acupuncture, such as floating acupuncture used in Ren et al.’s (26) study, may initiate a rapid onset of action for pain relief compared with SSRI/SNRI alone, and this may especially benefit patient with PSPD at their early stage of treatment. However, it was not possible to observe long-term effect of acupuncture and SSRI/SNRI for patients with SSD due to data availability.

### 4.1 Limitations

There are some limitations of this study that could affect the interpretation of the result: (1) the number of studies included in this review was very small with small sample size, and the overall quality of the studies included in this review was moderate to low; this largely undermined the confidence in the result; (2) due to absence of SSD treatment guideline or consensus, and characteristic of acupuncture practice, there is large heterogeneity of outcome indicators, acupuncture type and treatment protocol, study design, etc., which limited the synthesis of outcomes data; (3) follow-up periods of all studies were short, and the long-term effects could not be observed; (4) all included studies were from China, which may limit the extrapolation of the results; (5) the limited number of studies and their intervention design precluded further analyses to distinguish the effect between acupuncture combined with SSRIs and SNRIs in this review.

## 5 Conclusion

In conclusion, this study found that acupuncture combined with SSRI/SNRI may provide an incremental effect on anxiety for patients with SSD compared with medication alone at 4 weeks, but its effect on pain relief remained inconsistent across studies. Due to small number of studies included in this review, well-designed randomized controlled trials with satisfactory sample size are needed to further confirm the effect and safety of acupuncture for patients with SSD.

## Data Availability

The original contributions presented in the study are included in the article/Supplementary material, further inquiries can be directed to the corresponding authors.
