# Correction: Effect of acupuncture on somatic symptom disorder: a systematic review and meta-analysis

**DOI:** 10.3389/fmed.2025.1717418

**Published:** 2025-10-30

**Authors:** Feixue Zhao, Weiming Wang, Xinyao Feng, Jinyu Li, Weijuan Gang, Xianghong Jing, Huan Chen, Ying Liang

**Affiliations:** ^1^Peking University Sixth Hospital, Peking University, Beijing, China; ^2^Department of Acupuncture and Moxibustion, Guang'anmen Hospital, China Academy of Chinese Medical Sciences, Beijing, China; ^3^Institute of Acupuncture and Moxibustion, China Academy of Chinese Medical Science, Beijing, China; ^4^Key Laboratory of Mental Health, National Clinical Research Center for Mental Disorders, Ministry of Health, Institute of Mental Health, Beijing, China

**Keywords:** somatic symptom disorder, SSD, persistent somatoform pain disorder, PSPD, acupuncture, meta-analysis

In the published article, there was a mistake in the original text. The second paragraph of *3.4.1 HAMA* was displayed as “In Sun et al. (23) (*n* = 60) compared electroacupuncture combined with duloxetine (SNRI) to duloxetine alone; Liang and Liang (24) (*n* = 70) compared acupuncture combined with duloxetine (SNRI) to duloxetine alone; Ma et al. (25)'s study, the between-group difference in the change of HAMA scores after 6 weeks of treatment in the experimental group was significantly higher than that in the control group (−5.00, 95%CI: −7.80 to −2.20; p = 0.0009) from baseline; however, the mean difference was not significant at weeks 2 (0.30, 95%CI: −2.56 to 3.16; p = 0.84) and 4 (−2.3, 95%CI: −5.11 to 0.51; p = 0.11) (shown in Table 2).”

The correct text should read: “In Sun et al. (23)'s study, the between-group difference in the change of HAMA scores after 6 weeks of treatment in the experimental group was significantly higher than that in the control group (−5.00, 95% CI: −7.80 to −2.20; *p* = 0.0009) from baseline; however, the mean difference was not significant at weeks 2 (0.30, 95% CI: −2.56 to 3.16; *p* = 0.84) and 4 (−2.3, 95% CI: −5.11 to 0.51; *p* = 0.11) (shown in Table 2).”

The original version of this article has been updated.

