# Supplementary material for: Effect of acupuncture on somatic symptom disorder: a systematic review and meta-analysis
Source: Front Med (Lausanne). 2025 Oct 1;12:1625230. doi: 10.3389/fmed.2025.1625230 (PMC12521258; doi:10.3389/fmed.2025.1625230)
Supplement: Supplementary file 1 [file Data_Sheet_1.docx]

Supplementary Material

# Supplementary File S1. Search strategies for databases.

**PubMed**

A total of 64 articles were retrieved from the PubMed database on 15th March 2024, Beijing time.

Search strategies:

#1 ("Somatoform Disorders"[Mesh:NoExp]) OR ((((((((((((((((somatoform disorder) OR (somatization)) OR (somatisation)) OR (medically unexplained syndrome)) OR (medically unexplained symptom)) OR (Syndrome, Medically Unexplained)) OR (Unexplained Syndrome, Medically)) OR (psychogenic pain)) OR (pain disorder)) OR (somatoform pain)) OR (psychalgia)) OR (somatic symptom disorder)) OR (Briquet Syndrome)) OR (Syndrome, Briquet)) OR (bodily distress disorder)) OR (functional somatic disorder))

#2 ((electroacupuncture OR "acupuncture"[mesh terms] OR "acupuncture"[all fields] OR "acupuncture therapy"[mesh terms] OR "acupuncture therapy"[all fields] OR auricular acupuncture OR auricular needle OR ear acupuncture OR auricular plaster therapy OR transcutaneous electric nerve stimulation OR tens OR electric stimulation therapy OR laser acupuncture OR auricular point sticking OR acupressure OR dry needle OR scalp acupuncture OR scalp sensory OR scalp stimulation OR filliform needle OR filiform needle OR acupuncture analgesia OR electroanalgesia OR moxibustion OR pharmacopuncture OR pharmacoacupuncture))

#3 ((randomized controlled trial OR randomised controlled trial OR Controlled Clinical Trial OR placebo[Title/Abstract] OR sham[Title/Abstract] OR randomised[Title/Abstract] OR randomized[Title/Abstract] OR randomly[Title/Abstract] OR trial[Title/Abstract] OR groups[Title/Abstract])) not (animals NOT humans)

#4 #1 AND #2 AND #3

**Embase**

A total of 1,482 articles were retrieved from the Embase database on 15th March 2024, Beijing time.

Search strategies:

#1 'somatoform disorder'/exp OR 'somatoform disorder' OR 'somatization'/exp OR 'somati？ation' OR 'medically unexplained symptom'/exp OR 'medically unexplained symptom' OR 'medically unexplained syndrome' OR (medically AND unexplained AND ('symptom'/exp OR symptom OR 'syndrome'/exp OR 'syndrome')) OR 'psychogenic pain'/exp OR 'psychogenic pain' OR (psychogenic AND ('pain'/exp OR pain)) OR 'pain disorder' OR (('pain'/exp OR pain) AND disorder) OR 'somatoform pain' OR (somatoform AND ('pain'/exp OR pain)) OR psychalgia OR 'somatic symptom disorder' OR (somatic AND ('symptom'/exp OR symptom) AND disorder) OR 'briquet syndrome' OR (briquet AND ('symptom'/exp OR 'symptom')) OR 'bodily distress disorder' OR 'functional somatic disorder'

#2 'electroacupuncture'/exp OR electroacupuncture OR 'acupuncture therapy'/exp OR 'acupuncture therapy' OR (('acupuncture'/exp OR acupuncture) AND ('therapy'/exp OR therapy)) OR 'acupuncture moxibustion' OR 'acupuncture moxibustion'/exp OR 'acupuncture moxibustion' OR (('acupuncture'/exp OR acupuncture) AND ('moxibustion'/exp OR moxibustion)) OR 'auricular acupuncture'/exp OR 'auricular acupuncture' OR (auricular AND ('acupuncture'/exp OR acupuncture)) OR 'auricular needle'/exp OR 'auricular needle' OR (auricular AND ('needle'/exp OR needle)) OR 'ear acupuncture'/exp OR 'ear acupuncture' OR (('ear'/exp OR ear) AND ('acupuncture'/exp OR acupuncture)) OR 'auricular plaster therapy' OR (auricular AND ('plaster'/exp OR plaster) AND ('therapy'/exp OR therapy)) OR 'transcutaneous electric nerve stimulation'/exp OR 'transcutaneous electric nerve stimulation' OR (transcutaneous AND electric AND ('nerve'/exp OR nerve) AND ('stimulation'/exp OR stimulation)) OR tens OR 'electric stimulation therapy'/exp OR 'electric stimulation therapy' OR (electric AND ('stimulation'/exp OR stimulation) AND ('therapy'/exp OR therapy)) OR 'laser acupuncture'/exp OR 'laser acupuncture' OR (('laser'/exp OR laser) AND ('acupuncture'/exp OR acupuncture)) OR 'auricular point sticking' OR (auricular AND point AND sticking) OR 'acupressure'/exp OR acupressure OR 'dry needle' OR (dry AND ('needle'/exp OR needle)) OR 'scalp acupuncture'/exp OR 'scalp acupuncture' OR (('scalp'/exp OR scalp) AND ('acupuncture'/exp OR acupuncture)) OR 'scalp sensory' OR (('scalp'/exp OR scalp) AND ('sensory'/exp OR sensory)) OR 'scalp stimulation' OR (('scalp'/exp OR scalp) AND ('stimulation'/exp OR stimulation)) OR 'filiform needle' OR (filiform AND ('needle'/exp OR needle)) OR 'filiform needle' OR (filiform AND ('needle'/exp OR needle)) OR 'acupuncture analgesia'/exp OR (('acupuncture'/exp OR acupuncture) AND ('analgesia'/exp OR analgesia)) OR 'electroanalgesia'/exp OR electroanalgesia OR 'pharmacopuncture'/exp OR Pharmaco*puncture OR herb* AND ('acupuncture'/exp OR acupuncture)

#3 ('randomized controlled trial'/exp OR 'randomi？ed controlled trial' OR (randomi？ed AND controlled AND ('trial'/exp OR trial)) OR 'controlled clinical trial'/exp OR 'controlled clinical trial' OR (controlled AND ('clinical'/exp OR clinical) AND ('trial'/exp OR trial)) OR 'placebo'/exp OR placebo OR sham OR randomized OR randomly OR 'trial'/exp OR trial OR groups) AND 'human'/exp NOT 'animal'/de NOT 'rat'/exp NOT 'mouse'/exp

#4 #1 AND #2 AND #3

**Cochrane Library**

A total of 2,240 articles were retrieved from the Cochrane Library database on 15th March 2024, Beijing time.

Search strategies:

#1 Somatoform Disorders OR somatoform disorder OR somatization OR somatisation OR medically unexplained syndrome OR medically unexplained symptom OR (Syndrome, Medically Unexplained) OR (Unexplained Syndrome, Medically) OR psychogenic pain OR pain disorder OR somatoform pain OR psychalgia OR somatic symptom disorder OR Briquet Syndrome OR (Syndrome, Briquet) OR bodily distress disorder OR functional somatic disorder

#2 (electroacupuncture OR acupuncture OR auricular needle OR auricular plaster therapy OR transcutaneous electric nerve stimulation OR electric stimulation therapy OR auricular point sticking OR acupressure OR dry needle OR scalp sensory OR scalp stimulation OR filiform needle OR tens OR electroanalgesia OR moxibustion OR pharmacopuncture OR pharmacoacupuncture)

#3 (randomized controlled trial OR controlled clinical trial OR placebo OR sham OR randomized OR randomly OR trial OR groups) NOT (animal OR rat OR mouse)

#4 #1 AND #2 AND #3

**Web of Science (WoS)（Core Collection）**

A total of 995 articles were retrieved from the Wos database on 15th March 2024, Beijing time.

Search strategies:

#1 TS=Somatoform Disorders OR somatoform disorder OR somatization OR somatisation OR medically unexplained syndrome OR medically unexplained symptom OR (Syndrome, Medically Unexplained) OR (Unexplained Syndrome, Medically) OR psychogenic pain OR pain disorder OR somatoform pain OR psychhalgia OR somatic symptom disorder OR Briquet Syndrome OR (Syndrome, Briquet) OR bodily distress disorder OR functional somatic disorder

#2 TS=electroacupuncture OR acupuncture OR auricular needle OR auricular plaster therapy OR transcutaneous electric nerve stimulation OR electric stimulation therapy OR auricular point sticking OR acupressure OR dry needle OR scalp sensory OR scalp stimulation OR filiform needle OR tens OR electroanalgesia OR moxibustion OR pharmacopuncture OR pharmacoacupuncture

#3 TS=(randomized controlled trial OR controlled clinical trial OR placebo OR sham OR randomized OR randomly OR trial OR groups) NOT (animal OR rat OR mouse)

#4 #1 AND #2 AND #3

**China National Knowledge Internet (CNKI)**

A total of 29 articles were retrieved from the CNKI database on 15th March 2024, Beijing time.

Search strategies:

#1 SU=（‘躯体型障碍’ + ‘医学无法解释的症状’ + ‘Briquet综合征’ + ‘躯体化障碍’ + ‘疼痛障碍’ + ‘躯体痛苦障碍’ + ‘躯体形式障碍’ + ‘躯体症状障碍’） OR TI=（‘躯体型障碍’ + ‘医学无法解释的症状’ + ‘Briquet综合征’ + ‘躯体化障碍’ + ‘疼痛障碍’ + ‘躯体痛苦障碍’ + ‘躯体形式障碍’ + ‘躯体症状障碍’） OR KY=（‘躯体型障碍’ + ‘医学无法解释的症状’ + ‘Briquet综合征’ + ‘躯体化障碍’ + ‘疼痛障碍’ + ‘躯体痛苦障碍’ + ‘躯体形式障碍’ + ‘躯体症状障碍’）

#2 (SU=('针刺'+'电针'+'针灸'+'激光针'+'经皮电'+'经皮神经'+'电刺激'+'电止痛'+'体针'+'耳针'+'头针'+'毫针'+'干针'+'耳穴贴压'+'穴位按压'+'激光穴位照射'+'经皮电刺激治疗'+'经皮电刺激神经 '+'经皮电刺激'+'针刺治疗'+'针灸疗法'+'经皮神经电刺激'+'激光穴位'-'动物'-'鼠') OR TI=('针刺 '+'电针'+'针灸'+'激光针'+'经皮电'+'经皮神经'+'电刺激'+'电止痛'+'体针'+'耳针'+'头针'+'毫针'+' 干针'+'耳穴贴压'+'穴位按压'+'激光穴位照射'+'经皮电刺激治疗'+'经皮电刺激神经'+'经皮电 刺激'+'针刺治疗'+'针灸疗法'+'经皮神经电刺激'+'激光穴位'-'动物'-'鼠') OR KY=('针刺'+'电针 '+'针灸'+'激光针'+'经皮电'+'经皮神经'+'电刺激'+'电止痛'+'体针'+'耳针'+'头针'+'毫针'+'干针'+' 耳穴贴压'+'穴位按压'+'激光穴位照射'+'经皮电刺激治疗'+'经皮电刺激神经'+'经皮电刺激'+' 针刺治疗'+'针灸疗法'+'经皮神经电刺激'+'激光穴位'-'动物'-'鼠') OR AB=('针刺'+'电针'+'针 灸'+'激光针'+'经皮电'+'经皮神经'+'电刺激'+'电止痛'+'体针'+'耳针'+'头针'+'毫针'+'干针'+'耳穴 贴压'+'穴位按压'+'激光穴位照射'+'经皮电刺激治疗'+'经皮电刺激神经'+'经皮电刺激'+'针刺 治疗'+'针灸疗法'+'经皮神经电刺激'+'激光穴位'-'动物'-'鼠'))

#3 (SU='随机' or TI='随机' or KY='随机' or AB='随机')

#4 #1 AND #2 AND #3

**Wanfang Data（Includes English and Chinese extensions）**

A total of 39 articles were retrieved from the Wanfang database on 15th March 2024, Beijing time.

Search strategies:

#1 (题名或关键词:(“躯体型障碍” or “医学无法解释的症状” or “Briquet综合征” or “躯体化障碍” or “疼痛障碍” or “躯体痛苦障碍” or “躯体形式障碍” or “躯体症状障碍”) or 摘要:(“躯体型障碍” or “医学无法解释的症状” or “Briquet综合征” or “躯体化障碍” or “疼痛障碍” or “躯体痛苦障碍” or “躯体形式障碍” or “躯体症状障碍”))

#2 (题名或关键词:(“电针” OR “激光针” OR “经皮电” OR “经皮神经” OR “电刺激” OR “电 止痛” OR “体针” OR “耳针” OR “头针” OR “毫针” OR “干针” OR “耳穴贴压” OR “穴位 按压” OR “激光穴位照射” OR “tens” OR “镇痛皮肤电刺激” OR “针刺治疗” OR “针灸疗 法”) OR 摘要:(“电针” OR “激光针” OR “经皮电” OR “经皮神经” OR “电刺激” OR “电 止痛” OR “体针” OR “耳针” OR “头针” OR “毫针” OR “干针” OR “耳穴贴压” OR “穴位 按压” OR “激光穴位照射” OR “tens” OR “镇痛皮肤电刺激” OR “针刺治疗” OR “针灸疗 法”) OR 题名或关键词:(“针灸” OR “针刺”) OR 摘要:(“针灸” OR “针刺”))

#3 (题名 或关键词:“随机” OR 摘要:“随机”) NOT (题名或关键词:(“动物” OR “鼠”) OR 摘要:(“动 物” OR “鼠”))

#4 #1 AND #2 AND #3

**VIP Database for Chinese Technical Periodicals**

A total of 4,523 articles were retrieved from the Wanfang database on 15th March 2024, Beijing time.

Search strategies:

M=（躯体型障碍 OR 医学无法解释的症状 OR Briquet综合征 OR 躯体化障碍 OR 疼痛障碍 OR 躯体痛苦障碍 OR 躯体形式障碍 OR 躯体症状障碍） AND (电针 OR 激光针 OR 经皮电 OR 经皮电刺激治疗 OR 经皮电刺激神经 OR 经皮电刺激 OR 经皮神经 OR 电刺激 OR 电止痛 OR 体针 OR 耳针 OR 头针 OR 毫针 OR 干针 OR 耳穴贴压 OR 穴位按压 OR 激光穴位照射 OR “tens” OR 镇痛皮肤电刺激 OR 针刺 OR 针灸 OR 经皮神经电刺激 OR 激光穴位) AND (随机 NOT (动物 OR 鼠)) OR R=（躯体型障碍 OR 医学无法解释的症状 OR Briquet综合征 OR 躯体化障碍 OR 疼痛障碍 OR 躯体痛苦障碍 OR 躯体形式障碍 OR 躯体症状障碍） AND (电针 OR 激光针 OR 经皮电 OR 经皮电刺激治疗 OR 经皮电刺激神经 OR 经皮电刺激 OR 经皮神经 OR 电刺激 OR 电止痛 OR 体针 OR 耳针 OR 头针 OR 毫针 OR 干针 OR 耳穴贴压 OR 穴位按压 OR 激光穴位照射 OR “tens” OR 镇痛皮肤电刺激 OR 针刺 OR 针灸 OR 经皮神经电刺激 OR 激光穴位) AND (随机 NOT (动物 OR 鼠))

**China Biology Medicine disc (CBM)**

A total of 154 articles were retrieved from the CM database on 15th March 2024, Beijing time.

Search strategies:

#1【快速检索状态】：躯体型障碍 OR 医学无法解释的症状 OR Briquet综合征 OR 躯体化障碍 OR 疼痛障碍 OR 躯体痛苦障碍 OR 躯体形式障碍 OR 躯体症状障碍

#2【主题检索状态】：躯体型障碍 OR 医学无法解释的症状

#3 (#2) OR (#1)

#4【快速检索状态】：针刺 OR 电针 OR 耳针 OR 头针 OR 体针 OR 毫针 OR 针灸 OR

针灸疗法 OR 经皮神经电刺激 OR 经皮神经 OR 电刺激 OR 激光针 OR 耳穴贴压 OR 电止痛 OR 针刺镇痛 OR 干针 OR 穴位按压 OR 激光穴位照射 OR 针刺疗法 OR 电刺激疗法

#5【主题检索状态】：穴位, 耳针

#6【快速检索状态】：随机对照试验 OR 随机对照研究 OR 随机对照临床 OR 多中心研究 OR 多中心临床 OR 多中心

#7【快速检索状态】：动物 OR 大鼠 OR 小鼠 OR 鼠

#8 (#4 or #5) and #6

#9 (#4 or #5) and 文献类型限定（随机对照试验、多中心研究）

#10 (#8 or #9) not #7

#10 AND #3
