# Supplementary material for: Effect of acupuncture on somatic symptom disorder: a systematic review and meta-analysis
Source: Front Med (Lausanne). 2025 Oct 1;12:1625230. doi: 10.3389/fmed.2025.1625230 (PMC12521258; doi:10.3389/fmed.2025.1625230)
Supplement: Supplementary file 2 [file Data_Sheet_2.pdf]

Summary of findings:

Acupuncture+antidepressant compared to antidepressant for Somatic Symptom Disorder

Patient or population: Somatic Symptom Disorder
Setting: Studies conducted in hospital and outpatient settings
Intervention: acupuncture+antidepressant
Comparison: antidepressant

Outcomes	Anticipated absolute effects* (95% CI)		Relative effect (95% CI)	№ of participants (studies)	Certainty of the evidence (GRADE)	Comments
	**Risk with antidepressant**	**Risk with acupuncture+antidepressant**				
pooled HAMA - 2w	The mean pooled HAMA - 2w was **0**	MD **0.11 lower** (1.72 lower to 1.5 higher)	-	142 (2 RCTs)	⊕⊕◯◯ Low[a,b,c,d]	
pooled HAMA - 4w	The mean pooled HAMA - 4w was **0**	MD **1.94 lower** (3.71 lower to 0.17 lower)	-	142 (2 RCTs)	⊕⊕◯◯ Low[a,b,c,d]	
pooled HAMA - 6/8w	The mean pooled HAMA - 6/8w was **0**	MD **3.17 lower** (6.38 lower to 0.04 higher)	-	142 (2 RCTs)	⊕◯◯◯ Very low[a,b,c,d,e,f]	
pooled NRS - 2w	The mean pooled NRS - 2w was **0**	MD **1.25 lower** (3.03 lower to 0.53 higher)	-	154 (2 RCTs)	⊕◯◯◯ Very low[a,b,c,d,f]	
pooled NRS - 4w	The mean pooled NRS - 4w was **0**	MD **0.96 lower** (2.3 lower to 0.38 higher)	-	154 (2 RCTs)	⊕◯◯◯ Very low[a,b,c,d,f]	
pooled NRS - 6/8w	The mean pooled NRS - 6/8w was **0**	MD **1.27 lower** (3.81 lower to 1.26 higher)	-	154 (2 RCTs)	⊕◯◯◯ Very low[a,b,c,d,e,f]	
adverse effect assessed with: TESS or self report	No significant differences reported; mild symptoms resolved in both groups by 8 weeks.			(2 RCTs)	⊕⊕⊕◯ Moderate[a,b,c]	

***The risk in the intervention group** (and its 95% confidence interval) is based on the assumed risk in the comparison group and the **relative effect** of the intervention (and its 95% CI).

CI: confidence interval; **MD:** mean difference

GRADE Working Group grades of evidence
High certainty: we are very confident that the true effect lies close to that of the estimate of the effect.
Moderate certainty: we are moderately confident in the effect estimate: the true effect is likely to be close to the estimate of the effect, but there is a possibility that it is substantially different.
Low certainty: our confidence in the effect estimate is limited: the true effect may be substantially different from the estimate of the effect.
Very low certainty: we have very little confidence in the effect estimate: the true effect is likely to be substantially different from the estimate of effect.

Explanations

a. No allocation concealment
b. No blinding of participants and personnel
c. No blinding of outcome assessment
d. The sample size is small and the confidence interval is too wide.
e. Different time intervals
f. $I^2 > 50\%$, in the opposite direction
